# The effect of prepregnancy body mass index on maternal micronutrient status: a meta-analysis

**DOI:** 10.1038/s41598-021-97635-3

**Published:** 2021-09-13

**Authors:** Yan Yang, Zixin Cai, Jingjing Zhang

**Affiliations:** National Clinical Research Center for Metabolic Diseases, Metabolic Syndrome Research Center, Key Laboratory of Diabetes Immunology (Central South University), Ministry of Education, and Department of Metabolism and Endocrinology, The Second Xiangya Hospital of Central South University, Changsha, 410011 Hunan China

**Keywords:** Physiology, Diseases, Endocrinology, Health care

## Abstract

The relationship between prepregnancy body mass index (BMI) and maternal micronutrient status is inconsistent and has not received sufficient attention. This meta-analysis aimed to evaluate the effect of prepregnancy BMI on micronutrient levels in pregnant women. PubMed, Embase, Web of Science, and the Cochrane Library were searched for articles that contained information on micronutrient levels and prepregnancy BMI. A random-effects model was used to determine the association between prepregnancy BMI and maternal micronutrient status. Sixty-one eligible articles were eventually included, with 83,554 participants. Vitamin B12, folate, vitamin D, iron and ferritin were the main micronutrients evaluated in our meta-analysis. Prepregnancy obesity and overweight may lead to an increased risk of micronutrient deficiency, including vitamin B12, folate and vitamin D deficiency, while prepregnancy obesity or overweight may have no significant association with ferritin deficiency. Additionally, the results of the dose–response analyses demonstrated a possible significant inverse correlation between prepregnancy BMI and levels of micronutrient, except for iron and ferritin. Compared with women with normal weight, women who were overweight or obese prepregnancy have lower micronutrient concentrations and are more likely to exhibit micronutrient deficiency during pregnancy, which is harmful to both mothers and neonates.

## Introduction

Maternal micronutrients play an important role in the health of both mothers and infants^1,2^. For children, maternal micronutrient deficiency can result in perinatal morbidity and mortality and can even lead to chronic complications, such as metabolic syndrome, in adult life^1,3^. For mothers, lean birth can lead to an increased risk of pregnancy complications, including gestational diabetes mellitus and preeclampsia^2,4^.

Maternal obesity, defined as a body mass index (BMI) greater than 30 kg/m^2^^5^, is a major public health concern with an increasing prevalence worldwide^6^. Prepregnancy obesity has significant adverse effects on both mothers and offspring^7^. Obese women are more prone to experiencing stillbirth^8^, birth trauma^7^, gestational diabetes mellitus^9^ and preeclampsia^10^ than lean women. Additionally, adverse outcomes (e.g., preterm birth and congenital anomalies) are more common in infants of obese mothers^11,12^.

The micronutrient levels in the obese population are commonly ignored, particularly in pregnant women^13^. However, the consequences of maternal micronutrient deficiency are very harmful. Some of these adverse complications of obesity, such as preterm birth and congenital anomalies, have also been suggested to be related to maternal micronutrient status^11,14^. A report has demonstrated that vitamin D deficiency is common in obese women and increases the risk of food allergies^15^ and adiposity^16^ in offspring. Iron and ferritin may also be related to anthropometric results, while the exact connection is unknown. Increasing evidence has revealed a negative relationship between prepregnancy BMI and maternal micronutrition, mainly including vitamin B12, folate, vitamin D, iron and ferritin^17–20^; other studies have shown the opposite results^21–24^. Overall, the association between maternal micronutrition and obesity is unclear and remains to be studied. Given the inconsistent and ambiguous relationship between micronutrient levels and obesity in pregnant women, we conducted this meta-analysis to determine whether a higher prepregnancy BMI in mothers would lead to low micronutrient levels.

## Results

### Study characteristics

**Figure 1:**
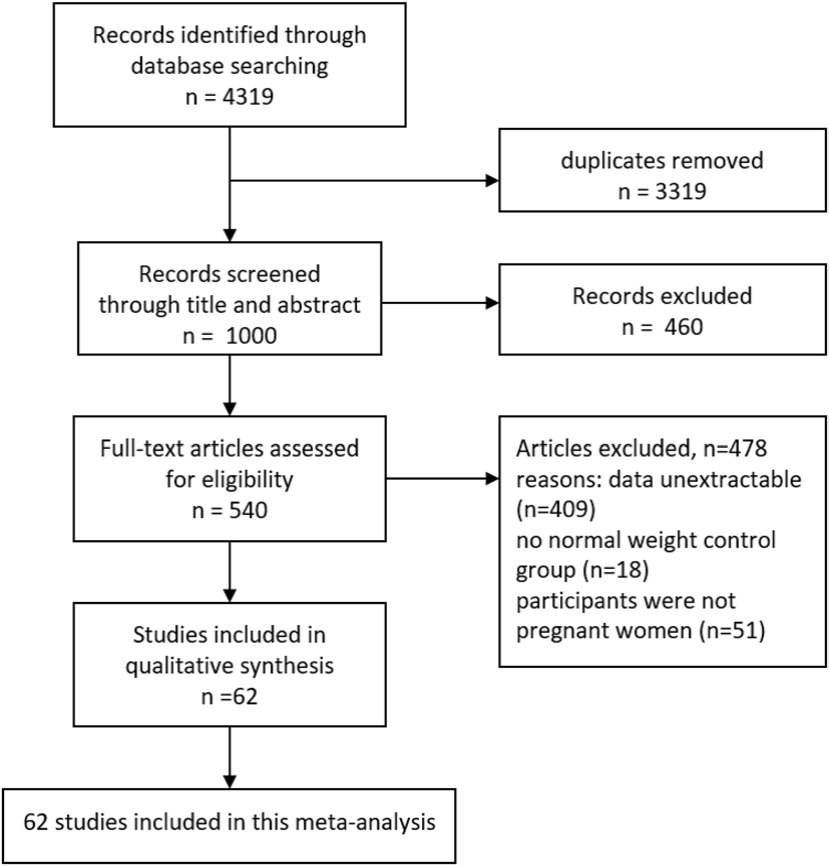
Flow diagram of the study selection process.

In total, 4319 studies were initially identified from 4 databases, including PubMed, the Web of Science, Embase and the Cochrane Library (Fig. 1). After removing duplicates, 1000 remaining studies were screened according to the titles and abstracts, and 460 studies were further excluded. Subsequently, 61 studies were selected after removing 487 studies according to the full-text screening. Finally, 61 articles^14,22–82^ were included in our meta-analysis. The main characteristics of the 61 included articles are shown in Table 1. Most of these articles were published between 2010 and 2020. Additionally, the definitions of micronutrient deficiency and methods to measure micronutrient status are listed in Table 2.

**Table 1:** Characteristics of the included studies.

No	Study	Year	Country	Type	Age	Measurement of BMI	Timing of micronutrient measurement	Timing of BMI measurement	n	Type of micronutrient	NOS
1	Adaikalakoteswari^25^	2015	UK	Cross-sectional	32.7 ± 5.9	Maternal recall	At 39–40 weeks of gestation	At the first pregnancy visit	91	Vitamin B12	7
2	Shuying LI^26^	2019	China	Cross-sectional	29.4 ± 4.5	NA	at 24–28 weeks of gestation	NA	406	Vitamin B12, folate	8
3	Riaz^23^	2018	Pakistan	Prospective	24.78 ± 4.89	Measured	≤ 13 weeks of gestation	Before pregnancy	301	Vitamin B12, folate, iron and vitamin D	6
4	Jun S. Lai^27^	2017	Singapore	Cross-sectional	NA	NA	At 26–28 weeks of gestation	Before pregnancy	913	Vitamin B12, folate	7
5	Peppard^28^	2019	USA	Cross-sectional	27	Measured	NA	Before pregnancy	174	Vitamin B12	8
6	Scholing^29^	2018	Netherlands	Cohort	30.9 ± 4.9	Maternal recall	At 12–15 weeks of gestation	At the first pregnancy visit	4243	Vitamin B12, folate, iron and ferritin	9
7	Monsen^22^	2016	Norway	Cohort	NA	Maternal recall	At 18 weeks of gestation	At the first pregnancy visit	2797	Vitamin B12, folate	8
8	Bhowmik^14^	2019	Bangladesh	Prospective	20.0 ± 2.6	Maternal recall	At 6–14 weeks of gestation	At the first pregnancy visit	498	Iron, ferritin, folate and B12	6
9	Shukri^30^	2015	UK	Case–control	NA	NA	At 16 and 28 weeks of gestation	Before pregnancy	241	Vitamin B12, folate and iron	6
10	Berglund^31^	2016	Spain	Cohort	Normal-weight = 30.9 ± 4.2, Overweight = 32.0 ± 4.2, Obese = 29.5 ± 7.8	NA	At 24 weeks of gestation	Before pregnancy	331	Vitamin B12, folate and ferritin	8
11	YS Han^32^	2011	Korea	Cross-sectional	Underweight = 30.7 ± 3.6, Normal-weight = 32.3 ± 4.0, Overweight = 32.8 ± 3.7, Obese = 32.9 ± 3.8	Maternal recall	At 19–39 weeks of gestation	At the first pregnancy visit	608	Folate	8
12	Minxue Shen^33^	2016	Canada	Cross-sectional	NA	Measured	At 12–20 weeks of gestation	NA	869	Folate	6
13	Tomedi^34^	2013	USA	Cohort	30.3 ± 5.6	MATERNAL recall	≤ 20 weeks of gestation	At the first pregnancy visit	129	Folate, vitamin D	8
14	Yamada^35^	2012	Japan	Cohort	38.5 ± 2.9	NA	At 5–13 weeks of gestation	Before pregnancy	5075	Folate	7
15	Santacruz^36^	2010	Spain	Cohort	Normal-weight = 31, Overweight = 29	Maternal recall	At 24 weeks of gestation	At the first pregnancy visit	50	Folate, iron	8
16	Shin^37^	2016	USA	Cross-sectional	NA	Maternal recall	NA	At the first pregnancy visit	795	Folate, iron	8
17	Abbas^38^	2017	Sudan	Cross-sectional	26.8 ± 6.2	Measured	< 14 weeks of gestation	NA	423	Iron	6
18	Chang Cao^24^	2015	USA	Cross-sectional	17.2 ± 1.1	Measured	At mid-gestation and/or at delivery	NA	230	Iron	9
19	Xiaobing Liu^39^	2017	China	Cross-sectional	27.0 ± 4.5	Measured	All trimesters	Before pregnancy	1400	iron	7
20	Raguž^40^	2016	Bosnia and Herzegovina	Cohort	29	NA	At delivery	Before pregnancy	128	Iron, ferritin	6
21	Lewandowska^41^	2020	Poland	Cohort	34.8 ± 4.4	Maternal recall	At 10–14 weeks of gestation	At the first pregnancy visit	563	Iron	9
22	Quijano^42^	2019	Mexico	Cohort	Adequate Weight = 22.71 ± 1.95, Obese = 34.81 ± 4.80	Maternal recall	At 13, 20, 27, and 34 weeks of gestation	At the first pregnancy visit	93	Iron, ferritin	9
23	Koenig^43^	2020	USA	Cross-sectional	27.6 ± 6.8	Maternal recall	At 29–33 weeks of gestation	At the first pregnancy visit	55	Iron, ferritin	8
24	Jones^44^	2016	China	Longitudinal study	Underweight = 24 ± 3.0, Normal-weight = 25 ± 3.5, Overweight = 26 ± 4.3, Obese = 25 ± 3.6	Maternal recall	At 24–28 weeks of gestation	At the first pregnancy visit	1613	Iron	7
25	Flynn^45^	2018	UK	Cohort	30 ± 4.2	NA	At 15–18 weeks of gestation	At the first pregnancy visit	490	Ferritin	7
26	Espı´nola^46^	2018	Spain	Cohort	Normal-weight = 31 ± 7, Overweight = 33 ± 4, Obese = 30.50 ± 8	NA	At 24–34 weeks of gestation	Before pregnancy	157	Iron	9
27	Lewicka^47^	2019	Poland	Cross-sectional	29.5 ± 4.8	Maternal recall	At delivery	At the first pregnancy visit	225	Iron	8
28	Mireku^48^	2016	Benin	Cohort	NA	NA	At the second trimester	Before pregnancy	636	Iron	7
29	Bodnar^49^	2004	USA	Cross-sectional	NA	Maternal recall	At 24–29 weeks of gestation	At the first pregnancy visit	439	Iron	6
30	Bener^50^	2013	Qatar	Cohort	NA	NA	Above 24 weeks of gestation	Before pregnancy	1873	Iron, vitamin D	6
31	COSTA^51^	2016		Cohort	31	NA	At 20 weeks of gestation	Before pregnancy		Iron	6
32	Figueiredo^52^	2019	Brazil	Cohort	26	Maternal recall	All trimesters	At the first pregnancy visit	163	Vitamin D	8
33	Nobles^53^	2015	USA	Cohort	18–40		At 15.2 weeks of gestation	Before pregnancy	237	Vitamin D	9
34	Yun^54^	2015	China	Cross-sectional	26.1	Maternal recall	NA	At the first pregnancy visit	1985	Vitamin D	7
35	Wang^55^	2019	China	Cross-sectional	Non-overweight and non-obesity = 28.8 ± 3.1, Overweight and obesity = 28.7 ± 3.2	Maternal recall	At 24–28 weeks of gestation	At the first pregnancy visit	140	Vitamin D	8
36	Chun^56^	2017	Korea	Cross-sectional	31.6	NA	At 3–17 weeks of gestation	Before pregnancy	356	Vitamin D	8
37	Yan Tian^57^	2016	USA	Cohort	NA	Maternal recall	At 4–29 weeks of gestation	At the first pregnancy visit	2558	Vitamin D	7
38	JM Thorp^58^	2012	USA	Case–control	Cases = 26.8 ± 5.5, Controls = 27.3 ± 5.6	Measured	At 16–21 weeks of gestation	Before pregnancy	265	Vitamin D	8
39	McAree^59^	2014	UK	Retrospective	NA	Measured	All trimesters	Before pregnancy	346	Vitamin D	6
40	Sen^60^	2017	USA	A secondary analysis of randomized controlled trial	28.4 ± 5.9	Measured	At 16 and 28 weeks of gestation	Before pregnancy	234	Vitamin D	7
41	Xin Zhao^61^	2017	China	Cohort	27.3 ± 3.9	Maternal recall	At 13 weeks of gestation	At the first pregnancy visit	13,806	Vitamin D	9
42	Rodriguez^62^	2016	Spain	Cohort	30.4 ± 4.3	Maternal recall	At 12 weeks of gestation	At the first pregnancy visit	2036	Vitamin D	9
43	Woon^63^	2019	Malaysia	Cohort	29.9 ± 4.1	Measured	Above 28 weeks of gestation	Before pregnancy	535	Vitamin D	7
44	Tuck^64^	2015	Australia	Cross-sectional	30.0 ± 5.4	Measured	At 12 weeks of gestation	Before pregnancy	1550	Vitamin D	7
45	Thiele^65^	2019	Portland	Cohort	30.6 ± 4.46	NA	Early pregnancy	At the first pregnancy visit	357	Vitamin D	8
46	Leffelaar^66^	2010	Netherlands	Cohort	≤ 24, 25–34, ≥ 35		Early pregnancy	At the first pregnancy visit	3730	Vitamin D	9
47	Choi^67^	2015	Korea	Cohort	32	Maternal recall	All trimesters	At the first pregnancy visit	220	Vitamin D	9
48	Eva Morales^68^	2014	Spain	Cohort	30.2 ± 4.6, 30.4 ± 4.3, 31.0 ± 4.2	Maternal recall	At 13–15 weeks of gestation	At the first pregnancy visit	2358	Vitamin D	8
49	Santos^69^	2018	Brazil	Cross-sectional	18–45	NA	Second or third trimester	At the first pregnancy visit	190	Vitamin D	9
50	Merewood^70^	2011	USA	Cross-sectional	< 20, 20–< 30, 30–43	Measured	Second or third trimester	Before pregnancy	459	Vitamin D	8
51	Karlsson^71^	2014	Sweden	Cross-sectional	Normal-weight = 31.4 ± 4.0 Obese = 32.0 ± 3.2	Maternal recall	First trimester	At the first pregnancy visit	105	Vitamin D	6
52	Burris^72^	2014	USA	Cohort	32.1 ± 5.0	NA	At 16.4–36.9 weeks of gestation	Before pregnancy	1591	Vitamin D	8
53	Huang^73^	2014	USA	Cohort	33.4 ± 4.2	Maternal recall	First trimester	At the first pregnancy visit	498	Vitamin D	9
54	Alonso^74^	2011	Spain	Cross-sectional	< 20, 20–29, ≥ 30	NA	First trimester	Before pregnancy	488	Vitamin D	6
55	Francis^75^	2018	USA	Cohort	28.2 ± 0.5	Maternal recall	At 10–14 and 15–26 weeks of gestation	At the first pregnancy visit	321	Vitamin D	8
56	Johns^76^	2017	USA	Cohort	18–24, 25–29, 30–34, ≥ 35	Measured	At 22.9–36.2 weeks of gestation	Before pregnancy	477	Vitamin D	6
57	Fernandez^77^	2014	USA	Cohort	15–24, 25–34, ≥ 35	Maternal recall	< 29 weeks of gestation	At the first pregnancy visit	2583	Vitamin D	8
58	López^78^	2013	Spain	Cross-sectional	< 20, 20–29, ≥ 30	NA	First trimester	Before pregnancy	502	Vitamin D	6
59	Woolcott^79^	2016	Canada	Case–control	< 25, 25–< 30, 30–< 35, ≥ 35	NA	At 20–28 weeks of gestation	Before pregnancy	1635	Vitamin D	8
60	Jani^80^	2020	Australia	Cohort	31.06 ± 5.176	Maternal recall	At 14 weeks of gestation	At the first pregnancy visit	16,528	Vitamin D	9
61	Daraki^81^	2018	Greece	Cohort	29.7 ± 4.9	NA	At 14 weeks of gestation	Before pregnancy	1226	Vitamin D	8

*NA* data not available, *NOS* Newcastle–Ottawa Scale.

**Table 2:** Characteristics of studies on micronutrient deficiency.

Study	Methods of micronutrient measurement	Definition of micronutrient deficiency
Musarrat Riaz (2018)^23^	ELISA/chemiluminescent immunoassay	Vitamin D deficiency (< 30 ng/ml) and low vitamin B12 (< 190 ng/l)
Bhowmik (2019)^14^	ELISA/chemiluminescent immunoassay	Vitamin D deficiency (< 30 nmol/l), vitamin B12 deficiency (< 200 pg/ml); folate deficiency (< 3 ng/ml) and iron deficiency (ferritin < 13 ng/ml)
Scholing (2018)^29^	Chemiluminescent immunoassay	Folate deficiency (< 10·0 nmol/l), iron deficiency (ferritin < 15·0 μg/l) and vitamin B12 deficiency (< 203·3 pg/ml)
Monsen (2016)^22^	Microbiological assay	NA
Abbas (2017)^38^	Radioimmunoassay gamma counter and kits	Iron deficiency (ferritin < 15 μg/l)
Chang Cao (2015)^24^	ELISA	Iron deficiency (ferritin < 12 μg/l)
Jones (2016)^76^	Chemiluminescent immunoassay	Iron deficiency (ferritin < 15 μg/l)
Koenig (2020)^43^	NA	Iron deficiency (ferritin < 12 μg/l)
Flynn (2018)^45^	ELISA	Iron deficiency (ferritin < 15 μg/l)
Bodnar (2004)^49^	NA	Iron deficiency (ferritin < 20 μg/l)
Nobles (2015)^53^	Heartland assays	25(OH)D < 20 ng/ml
Tomedi (2014)^83^	ELISA/chemiluminescent immunoassay	NA
Rodriguez (2016)^62^	BioRAD kit	25(OH)D < 20 ng/ml
Lo´pez (2011)^78^	Chemiluminescent immunoassay	25(OH)D < 20 ng/ml
Morales (2014)^68^	Chemiluminescent immunoassay	25(OH)D < 20 ng/ml
Thiele (2019)^65^	NA	25(OH)D < 29 ng/ml
Leffelaar (2010)^66^	ELISA	25(OH)D < 29.9 ng/ml
Daraki (2018)^81^	Chemiluminescent immunoassay	25(OH)D < 37.7 nmol/l
Choi (2015)^67^	NA	25(OH)D < 20 ng/ml
Santos (2017)^84^	Chemiluminescent immunoassay	25(OH)D < 50 nmol/l
Merewood (2010)^70^	Competitive protein-binding assay	25(OH)D < 20 ng/ml
McAree (2013)^59^	Liquid chromatography coupled to tandem mass spectrometry	25(OH)D < 25 nmol/l
Jani (2020)^80^	NA	25(OH)D < 50 nmol/l
TUCK (2015)^64^	Chemiluminescent immunoassay	25(OH)D < 50 nmol/l
Woolcott (2016)^79^	chemiluminescent immunoassay	25(OH)D < 50 nmol/l

*NA* data not available.

### Prepregnancy obesity (BMI ≥ 30) and micronutrient deficiency

**Figure 2:**
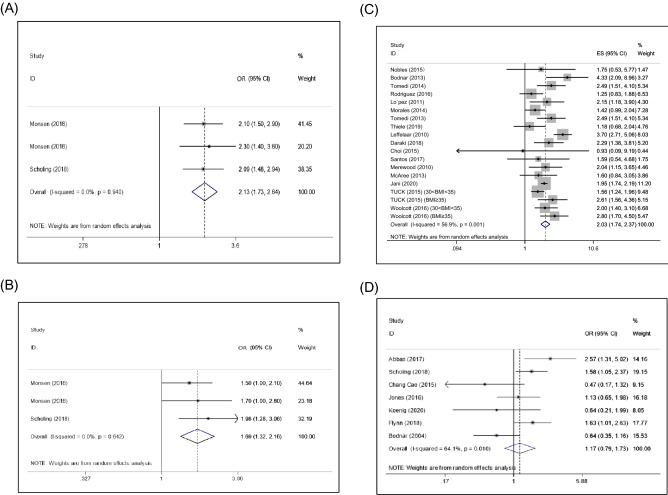
Forest plots of the relationship between prepregnancy obesity and micronutrient deficiency, including that of vitamin B12 (**A**), folate (**B**), vitamin D (**C**), and ferritin (**D**).

The pooled results from three included studies suggested that prepregnancy obesity (BMI > 30) contributed to an increased risk of vitamin B12 deficiency (OR: 2.13; 95% CI 1.73, 2.64) (Fig. 2A). Additionally, the overall data from three eligible studies showed that, compared with normal weight, prepregnancy obesity was positively associated with the prevalence of folate deficiency during pregnancy (OR: 1.69; 95% CI 1.32, 2.16) (Fig. 2B). The results in Fig. 2C from 17 studies demonstrate that prepregnancy obesity may be positively associated with the prevalence of vitamin D deficiency (OR: 2.03; 95% CI 1.74, 2.37). However, the data extracted from seven studies revealed that prepregnancy obesity may not be significantly associated with the risk of ferritin deficiency during pregnancy (OR: 1.17; 95% CI 0.79, 1.73) (Fig. 2D).

### Prepregnancy overweight (BMI: 25–29.9) and micronutrient deficiency

**Figure 3:**
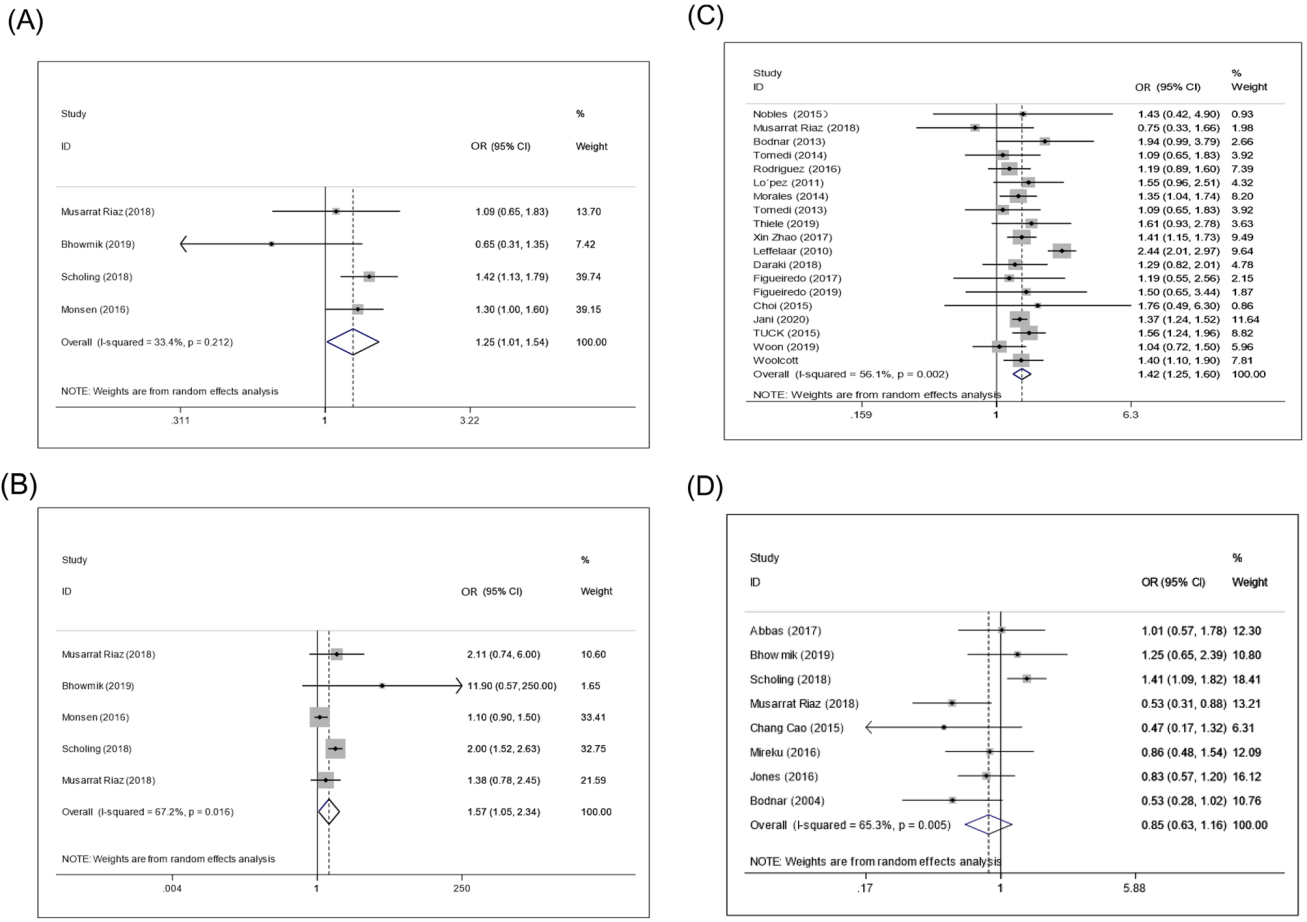
Forest plots of the relationship between prepregnancy overweight and micronutrient deficiency, including that of vitamin B12 (**A**), folate (**B**), vitamin D (**C**), and iron (**D**).

The pooled result from four included studies suggested that prepregnancy overweight contributed to an increased risk of vitamin B12 deficiency (OR: 1.25; 95% CI 1.01, 1.54) (Fig. 3A). The overall data extracted from nine eligible studies showed that, compared with normal weight, prepregnancy overweight was positively associated with the prevalence of folate deficiency during pregnancy (OR: 1.57; 95% CI 1.05, 2.34) (Fig. 3B). The overall data showed that, compared with normal weight, prepregnancy overweight was positively associated with the prevalence of vitamin D deficiency during pregnancy (OR: 1.42; 95% CI 1.25, 1.60) (Fig. 3C). Additionally, prepregnancy overweight may not be significantly associated with the risk of ferritin deficiency (OR: 0.85; 95% CI 0.63, 1.16) (Fig. 3D).

### Prepregnancy BMI and micronutrient level

**Figure 4:**
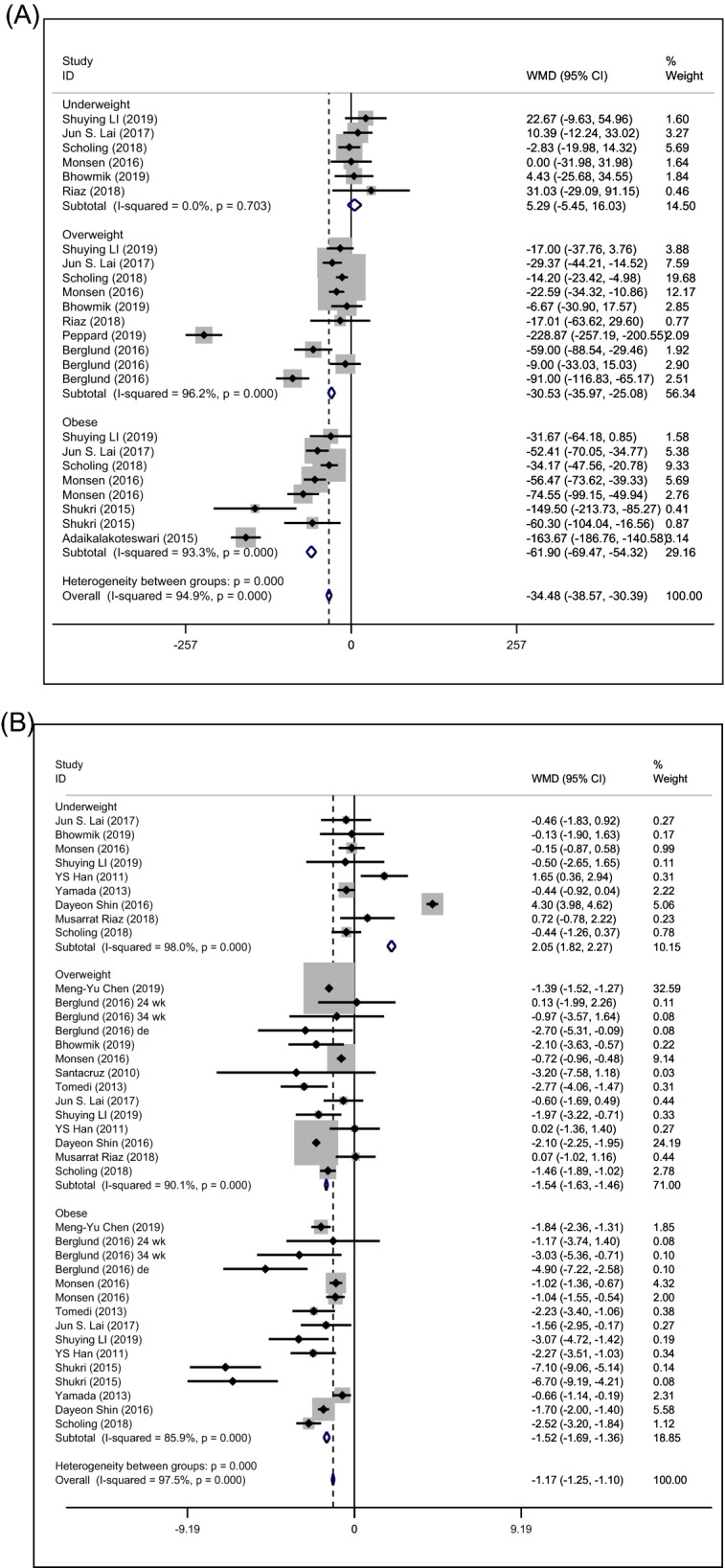

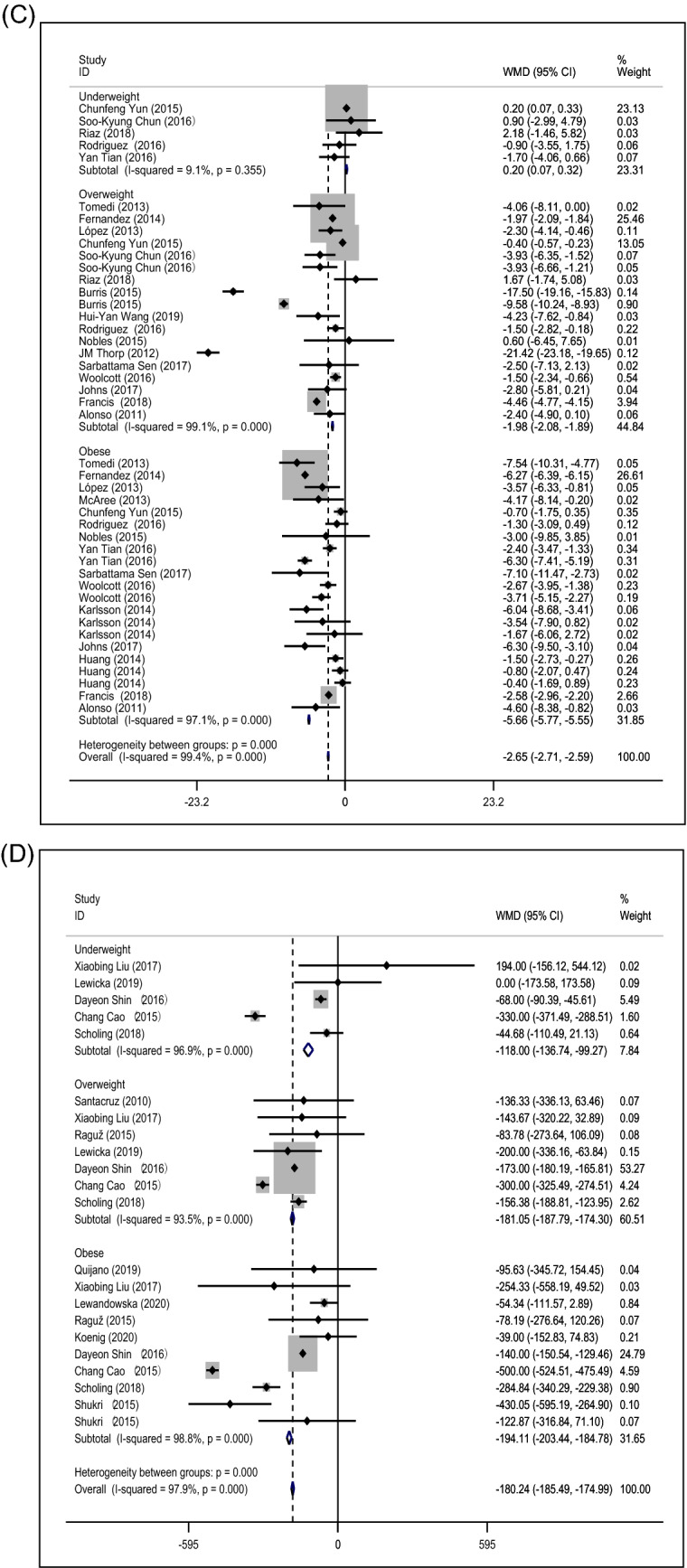

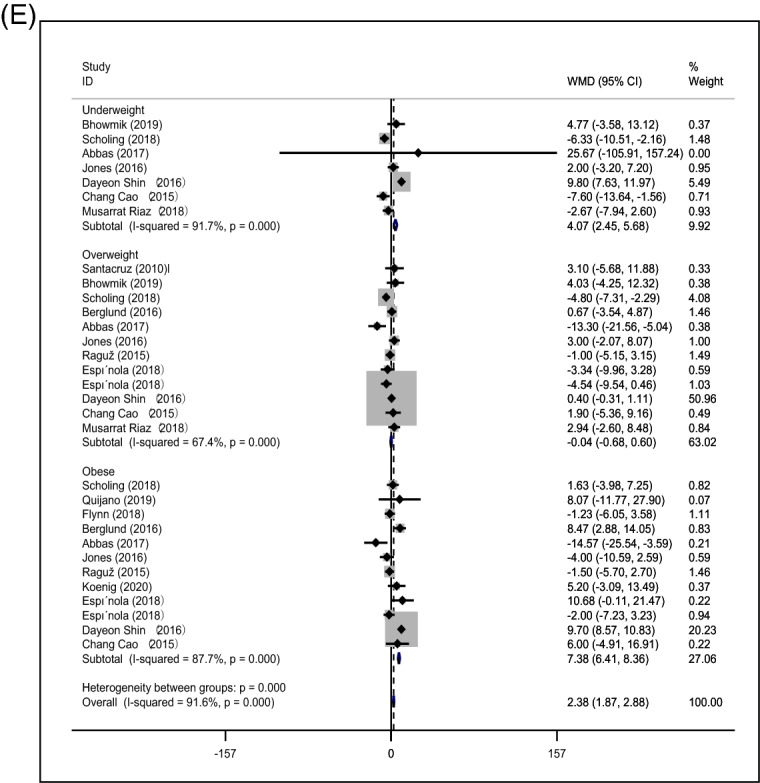
Forest plots between prepregnancy BMI and micronutrient deficiency, including vitamin B12 (**A**), folate (**B**), vitamin D (**C**), iron (**D**) and ferritin (**E**). Subgroup analysis of combined weighted mean differences with 95% confidence intervals was stratified by the prepregnancy BMI.

To further examine the relationship between prepregnancy BMI and vitamin B12, subgroup analysis based on prepregnancy BMI categories was conducted (Fig. 4A). The greatest decreases in vitamin B12 levels were observed in obese women (WMD: − 61.90 pg/ml; 95% CI [− 69.47, − 54.32]), followed by the overweight group (WMD: − 30.53 pg/ml; 95% CI [− 35.97, − 25.08]). However, prepregnancy underweight was not associated with maternal vitamin B12 levels (WMD: 5.9 pg/ml; 95% CI [− 5.45, 16.03]).

Second, subgroup analysis of the folate levels based on the prepregnancy BMI categories is shown in Fig. 4B. The greatest decreases in folate levels were observed in overweight women (WMD: − 1.52 ng/ml; 95% CI [− 1.69, − 1.36]) and the obese group (WMD: − 1.54 ng/ml; 95% CI [− 1.63, − 1.46]), while underweight prepregnancy may increase maternal folate levels (WMD: 2.05 ng/ml; 95% CI [1.82, 2.27]).

Third, the association of different prepregnancy BMI categories and vitamin D levels is revealed in Fig. 4C. Maternal vitamin D levels were significantly reduced in prepregnancy obese women (WMD: − 5.66 ng/ml; 95% CI [− 5.77, − 5.55]) and the overweight group (WMD: − 1.98 ng/ml; 95% CI [− 2.08, − 1.89]), while underweight prepregnancy may slightly increase maternal vitamin D levels (WMD: 0.20 ng/ml; 95% CI [0.007, 0.32]).

Additionally, the results of the association between different prepregnancy BMI categories and maternal iron were consistent (Fig. 4D). Compared with the normal-weight group, abnormal prepregnancy BMI decreased maternal iron levels (underweight WMD: − 118 µg/L; 95% CI [− 136.74, − 99.27]; overweight WMD: − 181.05 µg/L; 95% CI [− 187.79, − 174.30]; obese WMD: − 194.11 µg/L; 95% CI [− 203.44, − 184.78]).

However, as high heterogeneity existed in the above results (Fig. 4), we further conducted subgroup analysis based on methods for BMI measurement, timing of micronutrient measurement and timing of BMI measurement in underweight, overweight and obese women (Supplementary Tables 1–3). Although heterogeneity showed a certain degree of decline or increase, no true cause of heterogeneity can be fully identified, which may result from other information not provided in the included studies.

In contrast to iron, the association between prepregnancy BMI and serum ferritin was inconsistent. Prepregnancy underweight and obesity may be slightly related to the maternal ferritin level (underweight WMD: 4.07 µg/l, 95% CI [2.45, 5.66]; obese WMD: 7.36 µg/l, 95% CI [6.41, 8.36]), while overweight was not associated with ferritin level during pregnancy (WMD: − 0.04 ng/ml; 95% CI [− 0.68, 0.60]) (Fig. 4E).

### Dose–response analysis of prepregnancy BMI and micronutrients

**Figure 5:**
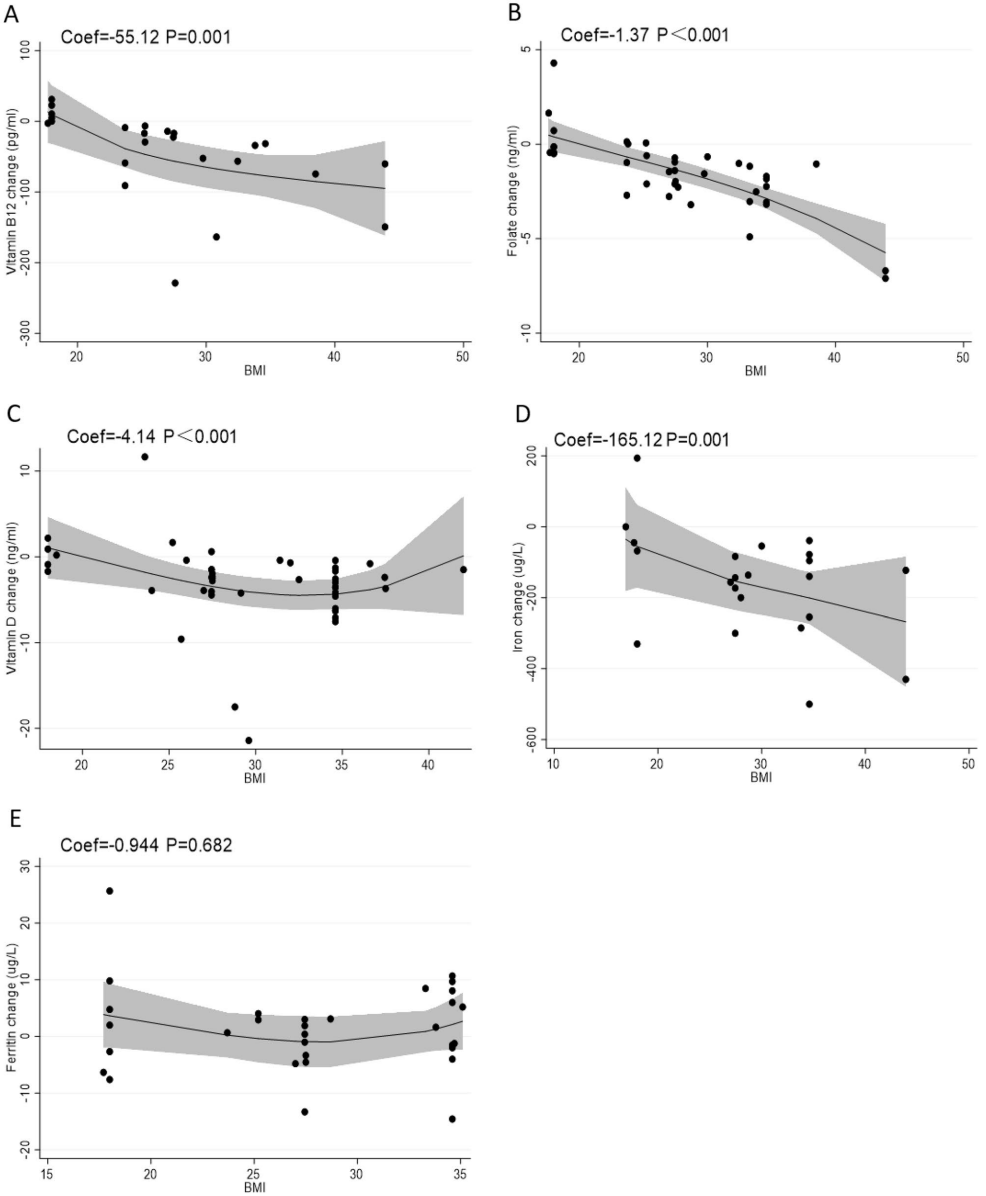
Nonlinear dose responses between prepregnancy BMI and micronutrient levels, including those of vitamin B12 (**A**), folate (**B**), vitamin D (**C**), iron (**D**) and ferritin (**E**).

Ten studies related to vitamin B12 were included; among them, 24 results were used to examine the dose–response relationship between prepregnancy BMI and vitamin B12. An inverse correlation was observed, as shown in Fig. 5A (coefficient =  − 55.12; P = 0.001).

Thirty-nine data points extracted from 15 studies demonstrated a significant inverse association between prepregnancy BMI and maternal folate (coefficient =  − 1.37; P < 0.001) (Fig. 5B).

The level of vitamin D was assessed by 25(OH) D measurement in the included articles to examine the association between prepregnancy BMI and vitamin D. Twenty-one studies were included in this analysis, and 45 results were extracted from the 21 studies. However, a significant inverse association was found between prepregnancy BMI and serum vitamin D (coefficient =  − 4.14; P < 0.001) (Fig. 5C).

Eleven studies and 20 subsequent data points revealed a significant inverse relationship between prepregnancy BMI and serum iron (coefficient =  − 165.12; P = 0.001) (Fig. 5D).

Fourteen studies were included, and 30 data points were extracted to examine the association between prepregnancy BMI and serum ferritin. No significant relationship was observed between prepregnancy BMI and serum ferritin (coefficient =  − 0.944; P = 0.682) (Fig. 5E).

### Evaluation of publication bias and sensitivity analysis

**Figure 6:**
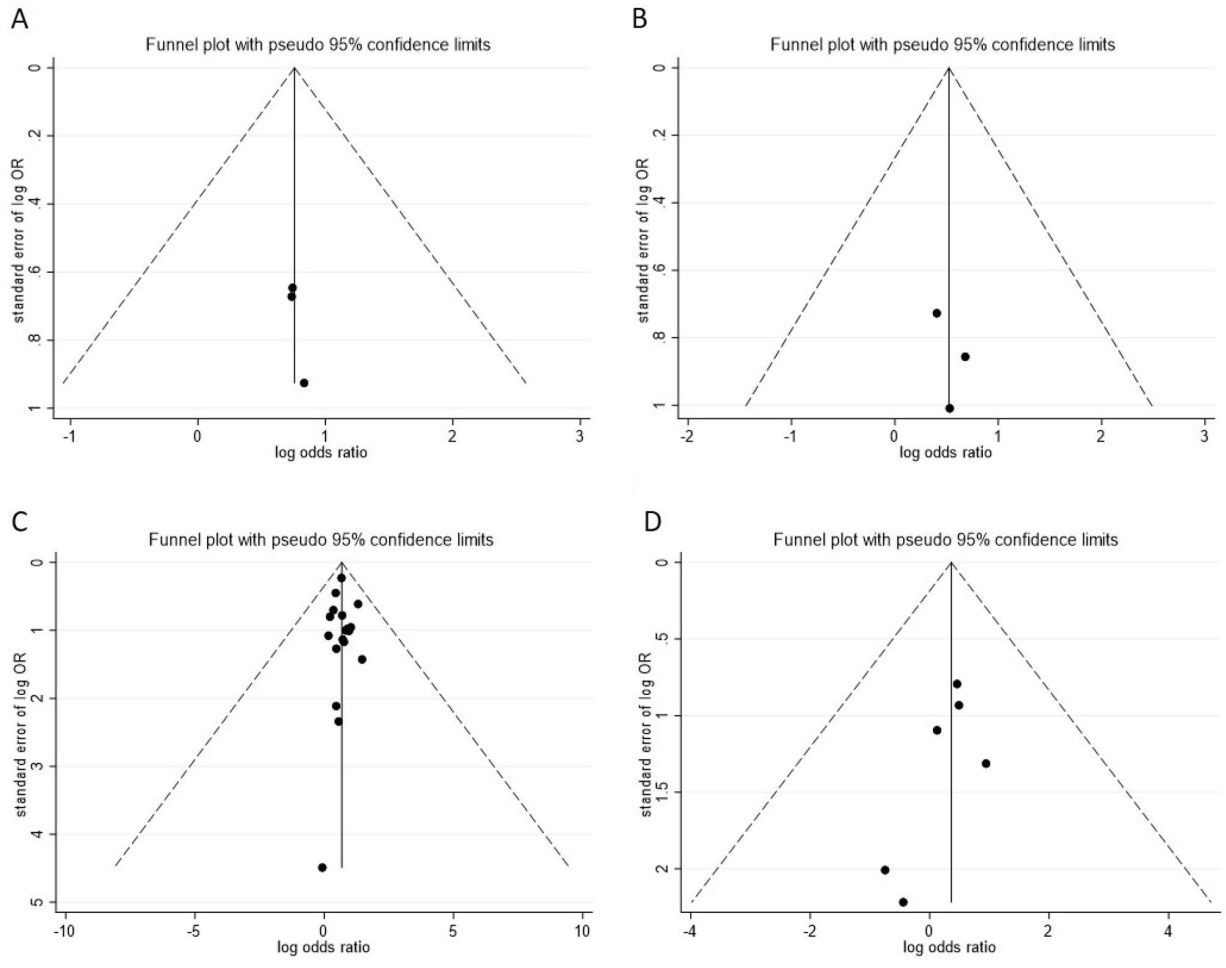
Funnel plots between prepregnancy obesity and micronutrient deficiency, including those of vitamin B12 (**A**), folate (**B**), vitamin D (**C**), and iron (**D**).

**Figure 7:**
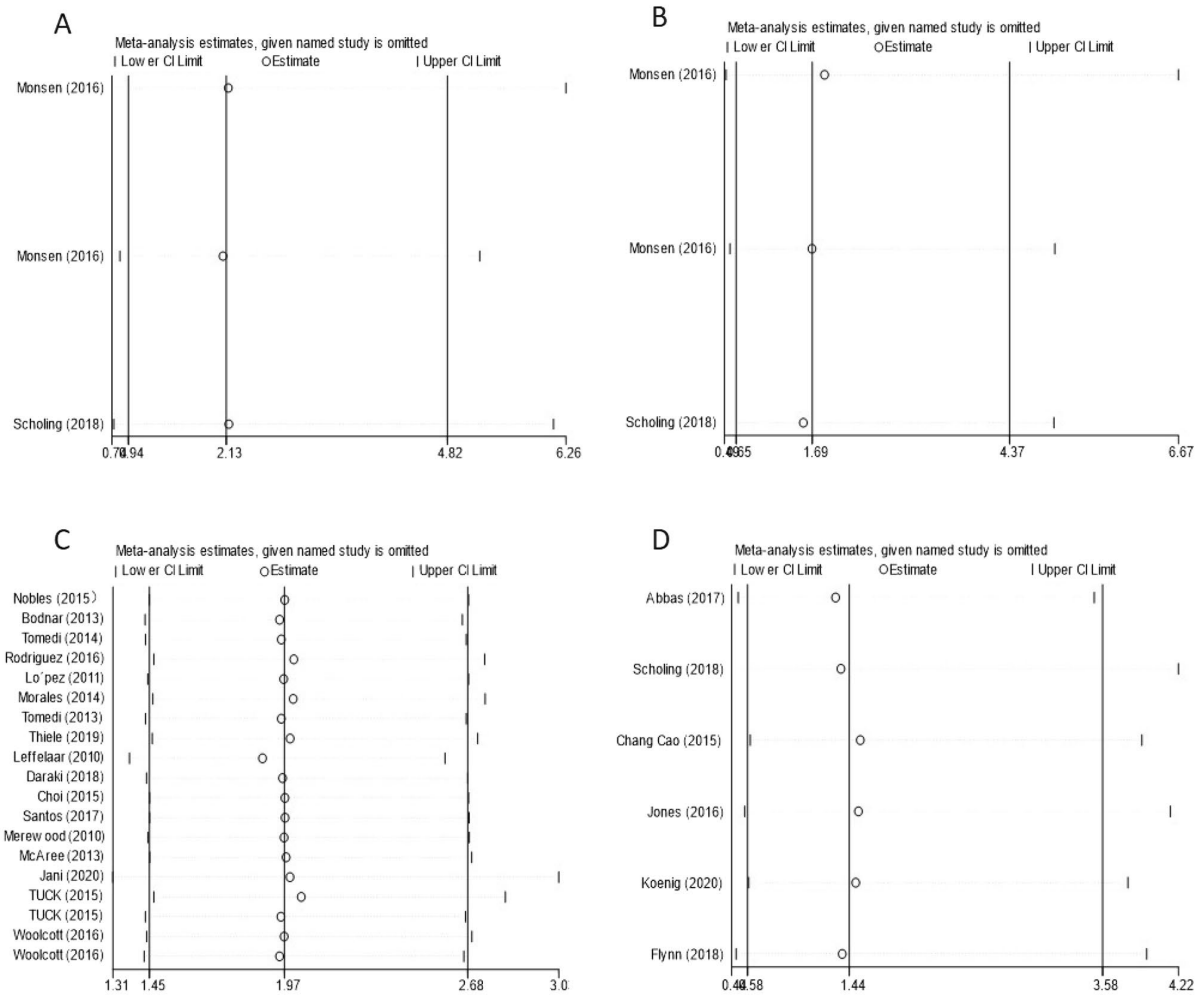
Sensitivity analysis between prepregnancy obesity and micronutrient deficiency, including that of vitamin B12 (**A**), folate (**B**), vitamin D (**C**), and iron (**D**).

Funnel plots, Egger’s regression test and Begg’s rank correlation test were used to analyse publication bias in our meta-analysis. The proportion of statistically significant publication bias tests was not observed for larger meta-analyses, as detected by either Begg’s or Egger’s test (P > 0.05). Funnel plots also showed symmetric distribution in every analysis (Fig. 6). Overall, no publication bias was found in our meta-analysis. Additionally, sensitivity analysis further demonstrated that our results were stable (Fig. 7).

## Discussion

Micronutrients play an important role in the health of mothers and offspring. The levels of micronutrients in the obese population, particularly in obese pregnant women, are usually neglected. However, recent studies have shown that an inverse relationship may exist between obesity and micronutrient levels^17,85^, while some studies have found the opposite relationship^23,24^. Therefore, we performed the present meta-analysis to resolve this discrepancy. To the best of our knowledge, this systematic review and meta-analysis is the first to assess the relationship between prepregnancy BMI and pregnancy micronutrient levels.

Our study mainly focused on five common micronutrients: vitamin B12, folate, vitamin D, iron and ferritin. Based on our findings from all 62 papers, micronutrient deficiencies, including those of vitamin B12, folate, and vitamin D, were more frequent in obese or overweight pregnant women than in nonobese women (Figs. 2 and 3). Additionally, we found a direct inverse association in pregnant women between prepregnancy BMI and maternal levels of micronutrients, except for ferritin (Figs. 4 and 5).

The aetiology of the inverse relationship between prepregnancy BMI and pregnancy micronutrient levels is unknown. Several factors may partially explain the link between BMI and maternal micronutrition. First, the consumption of a low-quality diet, characterized by less fruit and more calories, including solid fats, alcohol and added sugar^37^, may be an underlying mechanism. Obese people are more likely to consume a low-quality diet, which contributes to a lower intake of micronutrients before and during pregnancy than that of normal-weight women^37,86^.

Second, hepcidin, a marker of chronic inflammation in obesity^87^, may play a significant role in the association between prepregnancy BMI and iron. As an iron-regulating hormone^88,89^, hepcidin is increased in obese women, leading to reduced iron absorption and release^87^. Therefore, prepregnancy BMI may lead to a reduced level of iron in serum by inhibiting iron absorption.

Additionally, the lipid profile, a marker of obesity, is inversely associated with the level of vitamin B12 in T2DM patients^90^. Additionally, blood pressure and metabolic syndrome, complications of obesity, were accompanied by a low vitamin B12 status^91,92^. Thus, vitamin B12 may be reduced because of lipid disorders or complications of obesity.

Our meta-analysis has both practical and research implications. Regarding practical implications, we found that obese prepregnant women have a greater risk of micronutrient deficiency during pregnancy, indicating the importance of micronutrient supplementation and supervision in obese pregnant women. Additionally, we performed dose–response analyses to demonstrate the relationship between prepregnancy BMI and maternal micronutrient levels, including those of vitamin B12, folate, vitamin D, iron and ferritin. Finally, the relationship between prepregnancy obesity and micronutrients was systematically summarized in our study. Regarding research implications, identifying the underlying mechanisms of the effects of prepregnancy BMI on micronutrient deficiency may be an important direction of future research in this field to keep mothers and infants safe.

Although our study partially revealed the effects of obesity on pregnancy micronutrient levels, these levels were only measured during pregnancy and not before pregnancy in the included articles. Hence, future studies should include more details, such as prepregnancy micronutrient levels, to fully prove causality between BMI and pregnancy micronutrient levels. Additionally, high heterogeneity existed in our results. Information on the method and timing of BMI measurements, period of micronutrient measurement (Table 1) and definition of micronutrient deficiency (Table 2) were inconsistent, likely contributing to the high heterogeneity of our results. Furthermore, because some prepregnancy BMIs were obtained from maternal recall, which is not as accurate as the measured BMIs (Table 1), recall bias may exist in our analysis, and future clinical studies should focus more on the use of uniformly measured prepregnancy BMIs to avoid this bias. Moreover, the definition of micronutrient deficiency was not uniform in the different included papers (Table 2); for example, the different standards of deficiency are also a limitation, and more well-designed clinical studies are required. Additionally, as we did not add other iron biomarkers, including transferrin receptor and transferrin saturation, future meta-analyses to analyse the association between prepregnancy BMI and other iron levels are needed.

Finally, because micronutrient concentrations are often measured from plasma or serum, rather than whole blood, plasma volume changes during pregnancy can influence the concentrations of these micronutrients^93,94^. Therefore, new micronutrient cut-offs may be needed in future studies to avoid the possible effect of haemodilution in pregnant women. However, we focused on the relationship between prepregnancy BMI and maternal micronutrient levels, and the target population was pregnant women; thus, the effect of haemodilution may not affect our conclusion.

In conclusion, our study revealed that prepregnancy obesity or overweight may lead to an increased risk of micronutrient deficiency during pregnancy. Therefore, we emphasize that clinical micronutrient screening is necessary for overweight or obese pregnant women.

## Methods

### Search strategy

This meta-analysis was rigorously reported according to the Preferred Reporting Items for Systematic Reviews and Meta-Analyses (PRISMA) statement, as previously described^95^. This protocol analysis was registered on the PROSPERO website (protocol number: CRD42020188646). In this study, four electronic databases, PubMed, Embase, Cochrane Library and Web of Science, were searched for articles relevant to micronutrients and obesity through May 2020. The search terms were “BMI”, “obesity”, “overweight” and “body mass index” combined with “micronutrient”, “vitamin B12”, “folate”, “vitamin D”, “iron”, and “ferritin”. Additionally, we evaluated the references of the articles and reviews on micronutrients to identify studies that were not indexed in the databases but would be eligible for inclusion in this meta-analysis.

### Selection criteria

Two reviewers (YY and ZC) reviewed all the included studies and determined study eligibility. Disagreements were settled by consensus or the help of a third reviewer (JZ). All the articles included in this meta-analysis met the following criteria: (1) studies with information on obesity and micronutrients; (2) studies published in English; and (3) studies in which the micronutrients were limited to vitamin B12, folate, vitamin D, iron and ferritin. Additionally, articles were excluded if they met the following criteria: (1) articles that involved individuals who had undergone bariatric surgery; (2) articles that were literature reviews, communications or editorials; (3) studies with methodological weaknesses, such as inference data for the population from a nonrepresentative sample and studies that evaluated the relationship between prepregnancy BMI and nutritional status but did not explain the methodology or parameters used to evaluate these events; (4) studies in which data reported only in meeting abstracts would have been included if the abstract contained sufficient information for assessment; and (5) studies that did not have available information or usable data for this meta-analysis.

### Data extraction

All relevant articles were entered in EndNote X8 software and reviewed independently by two authors (YY and ZC). Discrepancies between authors were settled with the help of a third reviewer (JZ). The following information was extracted from the final studies: name of the first author, year of publication, country, sample size, study design, prepregnancy BMI, type of micronutrient, level of micronutrient, and odds ratio (OR) and 95% confidence interval (CI) of the micronutrient deficiency. All the extracted data were then imported into Excel software.

### Quality assessment of studies

The quality of the included studies was assessed using the Newcastle–Ottawa Scale (NOS)^96^. The measures on this scale comprise three items: the selection of participants, comparability of cases and controls, and ascertainment of outcomes. The scale has a minimum score of 0 and a maximum score of 9. Studies scoring at least 7 (corresponding to 78% of the maximum score) were regarded as having a low risk of bias (‘good’ quality), those scoring 4–6 were deemed to have a modest risk of bias (‘fair’ quality), and those scoring < 3 were considered to have a substantial risk of bias (‘poor’ quality)^97^. We assessed the quality of all the relevant studies in accordance with the type of study, sample size, participant selection, representativeness of the sample (case or exposure group), adequacy of follow-up, comparability (exposed-unexposed or case–control), and method of ascertainment for cases and controls. Finally, high-quality studies were included in the analyses. Two investigators (YY and ZC) independently performed the quality assessment. Any disagreements were settled with the help of a third reviewer (JZ) when necessary.

### Definition

Based on all the included studies, we classified BMI based on the World Health Organization (WHO) standards (underweight: BMI ≤ 18.5; normal weight: BMI 18.5–24.9; overweight: BMI 25–29.9; obesity: BMI ≥ 30). Doses (mean of BMI category) were defined as follows according to the data from the Scott‐Pillai study^98^: BMI 18.5–24.9 = 21.7; BMI 25–29.9 = 27.45; BMI 30–34.9 = 32.45; BMI 35–39.9 = 37.45; BMI ≤ 20 = 18.5; BMI < 25 = 21; BMI ≥ 25 = 30; BMI < 30 = 23.7; BMI ≥ 30 = 34.6; BMI ≥ 35 = 38.5; BMI ≤ 18.5 = 18 and BMI ≥ 40 = 41. Additionally, ferritin is an iron-storing protein, with serum ferritin regarded as a measurement of total body iron stores^99^. Furthermore, independent of iron status, serum ferritin is also increased by inflammation in the body because ferritin is an acute-phase protein^99^. To evaluate the potential dose–response relationship between BMI and micronutrient levels, a dose–response meta-analysis was conducted to compute the trend from the correlated values of BMI across various micronutrient levels.

### Statistical analysis

We gathered data on the prevalence of micronutrient deficiencies in various groups classified according to prepregnancy BMI. We gathered the results worldwide from different ethnicities and regions. Therefore, we used the random-effects model to obtain the meta-analysis results. Odds ratios (ORs) and CIs were used as summary measurements for the meta-analysis, and the results are presented as forest plots. Continuous variable effect size was defined as weighted mean differences (WMDs) and 95% CIs calculated for changes in micronutrient concentrations. Pooled WMDs with 95% CIs were calculated using the mean and standard deviation from each study by Stata 5 software. The correlation coefficient was used as another summary measure for the outcome studies, presented as dose response analyses. All statistical analyses were performed using Stata software (Version 13.0). The heterogeneity among all the studies was assessed by I^2^ statistics. The bias of the study was analysed using funnel plots. Sensitivity analysis was performed by leaving out each study one by one to evaluate the credibility of the pooled results.

## Supplementary Information

Supplementary Information 1.

Supplementary Information 2.

Supplementary Tables.
