# Supplementary material for: The effect of prepregnancy body mass index on maternal micronutrient status: a meta-analysis
Source: Sci Rep. 2021 Sep 13;11:18100. doi: 10.1038/s41598-021-97635-3 (PMC8437962; doi:10.1038/s41598-021-97635-3)
Supplement: Supplementary file 2 — Supplementary Information 2. [file 41598_2021_97635_MOESM2_ESM.docx]

**Search strategy**

1. ((BMI) AND pregnant) AND micronutrients
2. (((obese) OR body mass index) AND vitamin) AND pregnant
3. (((obese) OR overweight) AND vitamin B) AND pregnant
4. (((obese) OR overweight) AND vitamin D) AND pregnant
5. (((obese) OR overweight) AND iron) AND pregnant
6. (((obese) OR overweight) AND ferritin) AND pregnant
7. (((obese) OR body mass index) AND ((ferritin) OR (iron))) AND ((pregnant) OR (gestational))
8. (((obese) OR body mass index) AND ((25 (OH) D) OR (vitamin D))) AND ((pregnant) OR (gestational))
9. (“BMI” OR “obese” OR “body mass index” OR “obesity” OR “overweight”) AND (“micronutrient” OR “vitamin” OR “vitamin B12” OR “hydroxocobalamine” OR “folate” OR “vitamin D” OR “Fe” OR “iron” OR “ferritin”) AND (“gestational” OR “pregnancy”) AND (“case–control” OR “retrospective” OR “cohort” OR “cohorts” OR “prospective” OR “longitudinal” OR “follow-up” OR “cross-sectional” OR “trial”)
