# Supplementary material for: The effect of prepregnancy body mass index on maternal micronutrient status: a meta-analysis
Source: Sci Rep. 2021 Sep 13;11:18100. doi: 10.1038/s41598-021-97635-3 (PMC8437962; doi:10.1038/s41598-021-97635-3)
Supplement: Supplementary file 3 — Supplementary Tables. [file 41598_2021_97635_MOESM3_ESM.docx]

**Legend:**

**Supplementary table 1.** Subgroup analysis of micronutrients level in underweight pregnant women.

**Supplementary table 2.** Subgroup analysis of micronutrients level in overweight pregnant women.

**Supplementary table 3.** Subgroup analysis of micronutrients level in obese pregnant women.

**Supplementary table 1.** Subgroup analysis of micronutrients level in underweight pregnant women.

|  | Measurement of BMI | | | Timing of micronutrient measurement | | | Timing of BMI measurement | | |
| --- | --- | --- | --- | --- | --- | --- | --- | --- | --- |
|  | maternal recall | measured | NA | at first trimester | at second trimester | at third trimester | before pregnancy | at the first pregnancy visit | NA |
| **Folate** | | | | | | | | | |
| Comparisons | 5 | 1 | 3 | 3 | 3 | 3 | 2 | 5 | 2 |
| WMD | 1.07 (-1.49, 3.63) | 0.53 (-1.30, 2.36) | -0.45 (-0.89,0.00) | -0.28 (-0.80, 0.24) | -0.28 (-0.82, 0.26) | 0.33(-1.19, 1.85) | -0.09 (-1.13, 0.96) | 1.07 (-1.49, 3.63) | -0.47 (-1.63, 0.69) |
| I2 | 98.20% | - | 0.00% | 6.50% | 0.00% | 65.10% | 52.20% | 98.20% | 0.00% |
| P value of Q test | ＜0.001 | - | 0.999 | 0.343 | 0.59 | 0.057 | 0.148 | ＜0.001 | 0.973 |
| **Vitamin B12** | | | | | | | | | |
| Comparisons | 3 | 1 | 2 | 2 | 2 | 2 | 2 | 3 | 1 |
| WMD | -0.87 (-14.37, 12.64) | 31.03 (-29.09, 91.15) | 14.43 (-4.10, 32.97) | 9.77 (-17.16, 36.69) | -2.20 (-17.31, 12.91) | 14.43 (-4.10, 32.97) | 12.95 (-8.23, 34.13) | -0.87 (-14.37, 12.64) | 22.67 (-9.63, 54.96) |
| I2 | 0.00% | - | 0.00% | 0.00% | 0.00% | 0.00% | 0.00% | 0.00% | - |
| P value of Q test | 0.92% | - | 0.542 | 0.438 | 0.878 | 0.542 | 0.529 | 0.917 | - |
| **Vitamin D** | | | | | | | | | |
| Comparisons | 3 | 1 | 1 | 3 | 1 | 0 | 2 | 3 | 0 |
| WMD | -0.27 (-1.38,0.84) | 2.18 (-1.46, 5.82) | 0.90 (-2.99, 4.79) | 0.33 (-1.54, 2.21) | -1.70 (-4.06, 0.66) | - | -1.58 (-1.08, 4.24) |  | - |
| I2 | 36.10% | - | - | 0.00% | - | - | 0.00% |  | - |
| P value of Q test | 0.209 | - | - | 0.386 | - | - | 0.638 |  | - |
| **Iron** | | | | | | | | | |
| Comparisons | 3 | 2 | 0 | 1 | 1 | 1 | 1 | 3 | 1 |
| WMD | -64.62 (-85.66, -43.58) | -98.02 (-608.15, 412.11) | - | -330 (-371.49,-288.52) | -44.68 (-110.49, 21.13) | 0.00 (-173.58, 173.58) | 194.00 (-156.12, 544.12) | -64.62 (-85.66,-43.58) | -330 (-371.49,-288.51) |
| I2 | 0.00% | 88.20% | - | - | - | - | - | 0.00% | - |
| P value of Q test | 0.615 | 0.004 | - | - | - | - | - | 0.615 | - |
| **Ferritin** | | | | | | | | | |
| Comparisons | 4 | 3 | 0 | 3 | 1 | 1 | 1 | 4 | 2 |
| WMD | 2.56 (-6.06, 11.19) | -4.78 (-8.75, -0.81) | - | -0.11 (-5.49, 5.27) | -6.33 (-10.51, -2.16) | 2.00 (-3.20, 7.20) | -2.67 (-7.94, 2.60) | 2.56 (-6.06, 11.19) | -7.53 (-13.56, -1.5) |
| I2 | 93.70% | 0.00% | - | 14.20% | - | - | - | 93.70% | 0.00% |
| P value of Q test | ＜0.001 | 0.436 | - | 0.312 | - | - | - | ＜0.001 | 0.621 |

**Supplementary table 2.** Subgroup analysis of micronutrients level in overweight pregnant women.

|  | Measurement of BMI | | | Timing of micronutrient measurement | | | Timing of BMI measurement | | |
| --- | --- | --- | --- | --- | --- | --- | --- | --- | --- |
|  | maternal recall | measured | NA | at first trimester | at second trimester | at third trimester | before pregnancy | at the first pregnancy visit | NA |
| **Folate** | | | | | | | | | |
| Comparisons | 7 | 1 | 5 | 2 | 4 | 7 | 4 | 7 | 2 |
| WMD | -1.55 (-2.30, -0.79) | 0.07 (-1.02, 1.16) | -1.16 (-2.04, -0.28) | -0.95 (-3.07, 1.18) | -1.42 (-2.24, -0.61) | -0.98 (-1,79, -0.18) | -0.50 (-1,63, -0.62) | -1.55 (-2.30, -0.79) | -1.25 (-2.58, -0.09) |
| I2 | 94.20% | - | 25.50% | 80.50% | 87.80% | 29.90% | 26.90% | 94.20% | 61.30% |
| P value of Q test | ＜0.001 | - | 0.251 | 0.023 | ＜0.001 | 0.2 | 0.25 | ＜0.001 | 0.108 |
| **Vitamin B12** | | | | | | | | | |
| Comparisons | 3 | 2 | 5 | 2 | 3 | 5 | 6 | 3 | 1 |
| WMD | -16.52 (-23.47, -9.58) | -123.78 (-331.39, 83.83) | -40.08 (-66.47, -13.70) | -8.87 (-30.37, 12.64) | -17.58 (-25.65, -9.52) | -40.08 (-66.47, -13.70) | -72.63 (-134.41, -10.85) | -16.52 (-23.47, -9.58) | 17.00 (-37.76, 3.76) |
| I2 | 0.00% | 98.30% | 86.00% | 0.00% | 17.70% | 0.00% | 97.20% | 0.00% | - |
| P value of Q test | 0.385 | ＜0.001 | ＜0.001 | 0.7 | 0.27 | 0.08% | ＜0.001 | 0.385 | - |
| **Vitamin D** | | | | | | | | | |
| Comparisons | 6 | 4 | 8 | 7 | 4 | 7 | 12 | 6 | 0 |
| WMD | -2.45 (-3.84, -1.06) | -6.32 (-19.07, 6.42) | -5.29 (-9.41, -1.17) | -2.11 (-3.31, -0.92) | -3.35 (-5.57, -1.14) | -8.62 (-14.08, -3.15) | -5.62 (-9.58, -1.71) | -2.45 (-3.84, -1.06) | - |
| I2 | 99.10% | 98.60% | 98.40% | 40.70% | 99.10% | 99.00% | 98.50% | 99.10% | - |
| P value of Q test | ＜0.001 | ＜0.001 | ＜0.001 | 0.12 | ＜0.001 | ＜0.001 | ＜0.001 | ＜0.001 | - |
| **Iron** | | | | | | | | | |
| Comparisons | 3 | 2 | 1 | 3 | 1 | 2 | 2 | 3 | 1 |
| WMD | -172.18 (-179.19,-165.16) | -247.24 (-392.13,-102.36) | -83.78 (-273.64, -106.09) | -173.00 (-180.19, -165.81) | -156.38 (-188.81, -123.95) | -108.71 (-246.35, 28.92) | -115.89 (-245.18, 13.40) | -172.18 (-179.19, -165.16) | -300.00 (-325.49, -274.51) |
| I2 | 0.00% | 66.10% | - | 87.00% | - | 0.00% | 0.00% | 0.00% | - |
| P value of Q test | 0.581 | ＜0.001 | - | 0.05 | - | 0.709 | 0.651 | 0.581 | - |
| **Ferritin** | | | | | | | | | |
| Comparisons | 4 | 3 | 0 | 3 | 3 | 1 | 1 | 4 | 2 |
| WMD | 2.56 (-6.06, 11.19) | -4.78 (-8.75, -0.81) | - | -0.11 (-5.49, 5.27) | -6.33 (-10.51, -2.16) | 2.00 (-3.20, 7.20) | -2.67 (-7.94, 2.60) | 2.56 (-6.06, 11.19) | -7.53 (-13.56, -1.50) |
| I2 | 93.70% | 0.00% | - | 14.20% | 85.00% | - | - | 93.70% | 0.00% |
| P value of Q test | ＜0.001 | 0.436 | - | 0.312 | 0.003 | - | - | ＜0.001 | 0.621 |

**Supplementary table 3.** Subgroup analysis of micronutrients level in obese pregnant women.

|  | Measurement of BMI | | | Timing of micronutrient measurement | | | Timing of BMI measurement | | |
| --- | --- | --- | --- | --- | --- | --- | --- | --- | --- |
|  | maternal recall | measured | NA | at first trimester | at second trimester | at third trimester | before pregnancy | at the first pregnancy visit | NA |
| **Folate** | | | | | | | | | |
| Comparisons | 6 | 0 | 8 | 2 | 4 | 8 | 6 | 6 | 2 |
| WMD | -1.67 (-2.16, -1.18) | - | -3.43 (-5.23, -1.64) | -0.66 (-1.14, -0.19) | -1.61 (-2.34, -0.88) | -3.65 (-5.13, -2.17) | -3.88 (-6.55, -1.20) | -1.67 (-2.16, -1.18) | -2.24 (-3.71, -0.77) |
| I2 | 79.00% | - | 90.50% | - | 83.60% | 79.90% | 92.90% | 79.00% | 46.80% |
| P value of Q test | ＜0.001 | - | ＜0.001 | - | ＜0.001 | ＜0.001 | ＜0.001 | ＜0.001 | 0.171 |
| **Vitamin B12** | | | | | | | | | |
| Comparisons | 4 | 0 | 4 | 0 | 5 | 3 | 2 | 4 | 2 |
| WMD | -81.68 (-133.55, -29.82) | - | -63.55 (-96.52, -30.57) | - | -64.19 (-88.71, -39.66) | -82.92 (-162.50, -3.34) | -101.67 (-188.86, -14.49) | -81.68 (-133.55, -29.82) | -31.67 (-64.18, 0.85) |
| I2 | 96.70% | - | 71.20% | - | 79.10% | 97.10% | 80.20% | 96.70% | - |
| P value of Q test | ＜0.001 | - | 0.015 | - | 0.001 | ＜0.001 | 0.024 | ＜0.001 | - |
| **Vitamin D** | | | | | | | | | |
| Comparisons | 12 | 3 | 5 | 9 | 7 | 4 | 8 | 13 | 0 |
| WMD | -3.11 (-4.73, -1.50) | -5.86(-8.03, -3.70) | -3.25 (-4.12, -2.38) | -2.12 (-3.26, -0.99) | -4.90 (-7.13, -2.67) | -4.14 (-5.79, -2.49) | -3.71 (-4.60, -2.81) | -3.11 (-4.73, -1.50) | - |
| I2 | 98.20% | 0.00% | 97.10% | 61.90% | 98.90% | 58.60% | 8.20% | 98.20% | - |
| P value of Q test | ＜0.001 | 0.786 | ＜0.001 | 0.007 | ＜0.001 | 0.065 | 0.366 | ＜0.001 | - |
| **Iron** | | | | | | | | | |
| Comparisons | 6 | 2 | 3 | 3 | 2 | 5 | 4 | 6 | 1 |
| WMD | -142.27 (-215.92, -68.61) | -425.77 (-646.87, -204.66) | -215.86 (-443.51, 11.79) | -317.75 (-712.09, 76.59) | -169.70 (-395.58, 56.18) | -173.00 (-311.74, -34.25) | -224.30 (-404.65, -43.95) | -142.27 (-215.92, -68.61) | -500.00 (-524.51, -475.49) |
| I2 | 87.00% | 59.90% | 77.90% | 89.90% | 96.90% | 74.60% | 66.90% | 87.00% | - |
| P value of Q test | ＜0.001 | 0.114 | 0.011 | 0.002 | ＜0.001 | 0.003 | 0.029 | ＜0.001 | - |
| **Ferritin** | | | | | | | | | |
| Comparisons | 5 | 2 | 5 | 2 | 2 | 6 | 4 | 6 |  |
| WMD | 3.85 (-2.59, 10.28) | -4.27 (-24.43, 15.88) | 1.89 (-2.69, 6.47) | 6.48 (-3.08, 16.04) | -0.02 (-3.68, 3.64) | 2.05 (-2.50, 6.60) | 3.00 (-2.98, 8.98) | 2.84 (-3.24, 8.92) | -4.27(-24.43, 15.88) |
| I2 | 83.20% | 85.30% | 70.10% | 0.00% | 0.00% | 68.30% | 75.90% | 87.60% | 85.30% |
| P value of Q test | ＜0.001 | ＜0.001 | 0.009 | 0.858 | 0.448 | 0.008 | 0.006 | ＜0.001 | ＜0.001 |
